# Case report: Transcatheter closure of a giant and tortuous right coronary artery to right ventricle fistula in an infant

**DOI:** 10.3389/fcvm.2022.898914

**Published:** 2022-08-08

**Authors:** Yen-Hsien Wu, Yi-Ching Liu, Min-Fang Chao, Zen-Kong Dai, I-Chen Chen, Shih-Hsing Lo, Jong-Hau Hsu

**Affiliations:** ^1^Department of Pediatrics, Kaohsiung Medical University Hospital, Kaohsiung, Taiwan; ^2^Department of Medical Imaging, Kaohsiung Medical University Hospital, Kaohsiung, Taiwan; ^3^Department of Pediatrics, School of Medicine, College of Medicine, Kaohsiung Medical University, Kaohsiung, Taiwan

**Keywords:** coronary artery fistula (CAF), transcatheter closure (TCC), ADO II, pediatric (infant), right ventricle (RV)

## Abstract

Congenital coronary artery fistulas (CAFs) are an uncommon congenital anomaly. While most patients are asymptomatic, life-threatening events including sudden death, myocardial ischemia, heart failure, infective endocarditis, and rupture of aneurysm may occur. Surgical ligation was once the standard choice of management of CAFs in the past. However, transcatheter closure of CAFs has become an emerging alternative to surgery in patients with suitable anatomy. We reported a 7-month-old infant with a giant and tortuous CAF that originated from the distal right coronary artery and drained into the right ventricle, and was successfully treated by transcatheter closure with an Amplatzer ductus occluder.

## Introduction

Congenital coronary artery fistulas (CAFs) are abnormal connections between either or both coronary arteries and a cardiac chamber or a great vessel. It is a rare disease, and the incidence is around 0.002% of the general population (1). The natural history of CAFs is highly variable depending on their size. Those with small CAFs are often asymptomatic with incidental heart murmurs. Spontaneous closure has also been reported (2). However, patients with large CAFs may be complicated by acute myocardial ischemia, heart failure, infective endocarditis, and cardiac tamponade if there is a rupture of the fistula (3).

Coronary artery fistulas can be classified according to their origin or complexity. Sakakibara et al. suggested that a CAF can be classified as a proximal or distal type based on its origin (4). In the proximal type, the proximal native feeding arteries tend to be dilated, while the coronary arteries distal to the CAF remain normal. In contrast, in the distal type, the entire vessel is dilated. Based on the complexity of the morphology, a simple CAF has a single origin and drainage site through a single fistulous tract, whereas a complex CAF is composed of multiple origins or drainage sites with multiple fistulous structures (5).

Conventionally, surgical ligation was the standard option for management. Nevertheless, transcatheter closure of CAFs has recently become a potential alternative (3). In general, the favorable anatomies for transcatheter closure of CAFs include non-tortuous vessels, a single narrow drainage site, and a proximal origin. Herein, we report a 7-month-old infant with a large coronary artery (RCA) to the right ventricle (RV) fistula with a distal origin and tortuous right coronary artery. In this infant, the CAF was successfully obliterated by transcatheter closure with an Amplatzer ductus occluder II device.

## Case report

**FIGURE 1:**
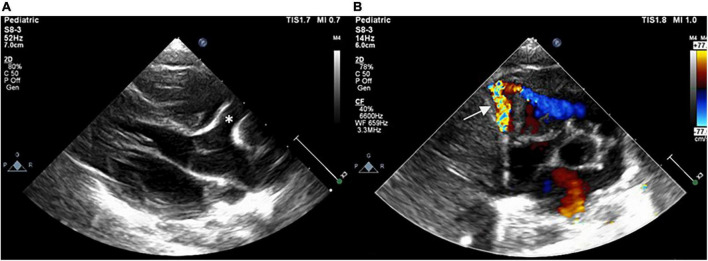
Postnatal echocardiogram showing dilated RCA (asterisk) with a fistula (arrow) drained into the RV in **(A)** parasternal long-axis view and **(B)** short-axis view. RCA, right coronary artery; RV, right ventricle.

**FIGURE 2:**
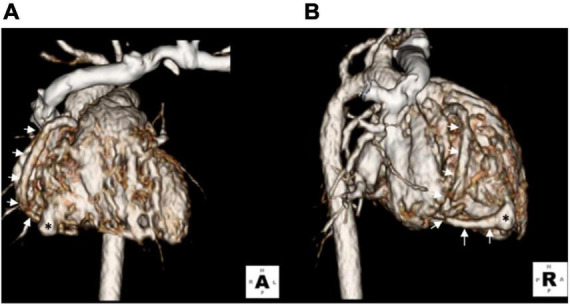
Three-dimensional computed tomography showing a lageniform coronary artery fistula (asterisks) arising from the RCA (arrows) drained into the inferior wall of the RV. **(A)** Anterior-posterior view. **(B)** Right lateral view.

A term female neonate was brought to our newborn intensive care unit with mild tachypnea 1 week after birth and a grade 4/6 continuous heart murmur in the left upper sternal border. Oxygen saturation was 100% under nasal cannula with a 1 L/min oxygen supply. Laboratory data revealed elevated serum B-type natriuretic peptide (BNP) (695 pg/ml) and normal troponin-I level (0.01 ng/ml). Electrocardiography showed sinus tachycardia with no evidence of myocardial ischemia, and her chest x-ray showed mild cardiomegaly with a cardiothoracic ratio of 0.58. Echocardiogram revealed a giant right coronary RCA to RV fistula with normal left ventricular systolic function. The orifice of the fistula on the RV side was about 2 mm (Figure 1). The fistula was found with a lageniform aneurysm arising from the distal RCA drained into the inferior wall of RV in the computed tomography angiography (CTA; Figure 2). Oral medications including furosemide and digoxin were administered, and the condition of congestive heart failure improved.

**FIGURE 3:**
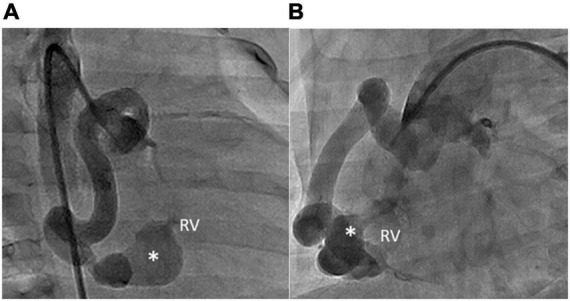
RCA angiography revealing that the aneurysmal fistula (asterisks) had arisen from the distal RCA and drained into the inferior wall of the RV. The shape of the fistula was lageniform. The aneurysmal tract of the fistula was 6 mm wide. The narrowest diameter of the drainage site was 1.5 mm. **(A)** RAO 30° and **(B)** LAO 60°.

**FIGURE 4:**
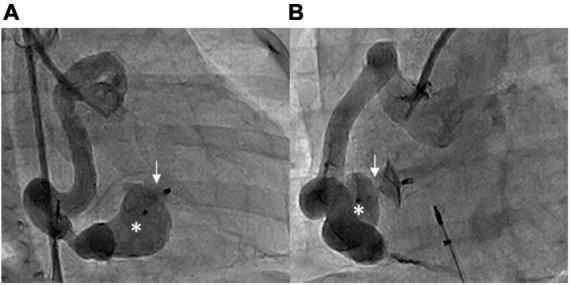
Post-procedural RCA angiography showing that the ADO II device (arrows) occluded the fistula with minimal residual shunt, with one disk in the aneurysmal tract (asterisks). **(A)** RAO 30° and **(B)** LAO 60°.

However, due to mild tachypnea and poor body weight gain (6.7 kg, 3 percentile), transcatheter closure of the fistula was performed at the age of 7 months old. The Qp/Qs was 1.2 while performing cardiac catheterization. However, a high mean pulmonary artery pressure of 23 mmHg was measured. RCA angiography revealed a giant and tortuous fistula that originated from the distal RCA and drained into the inferior RV, with an aneurysmal tract proximal to the drainage site (Figure 3). The size of the aneurysm was 6 mm, and the narrowest diameter of the drainage site was 1.5 mm. After delineating the morphology of the CAF, a 0.025-inch, 260-cm Terumo guidewire was first inserted from the RCA through the tortuous fistula to the RV, and then it was advanced to the pulmonary artery and snared to establish an arteriovenous wire loop. Based on the morphology of the aneurysmal tract and the width of the drainage site (1.5 mm), the Amplatzer Duct Occluder II (ADO II, 9-PDA2-04-04) with a 4-mm waist was chosen and the strategy for sizing the device for closure of patent ductus arteriosus was followed, that is, the occluder should be at least 2 mm greater than the narrowest drainage site. The ADO II device was then deployed through a low-profile 4 French delivery sheath and placed to occlude the fistula by antegrade approach from the femoral vein into the RV. After implantation of the device into the fistula and before releasing the device, a repeated RCA angiogram revealed a good device position with a minimal residual shunt. No ST segment change was noted in ECG, and no native coronary artery was involved by the device. After confirming the device’s proper size and positioning, the ADO II was released smoothly (Figure 4). No complications such as heart ischemia and atrioventricular block were noted. The post-procedural echocardiogram showed a proper position of the device without a residual shunt. The patient was discharged the next day with medications of aspirin (5 mg/kg/day) and clopidogrel (1 mg/kg/day) for 12 months. The follow-up CTA 12 months after the procedure showed good device position without residual shunt or recanalization of the fistula. There was a slow flow state in the aneurysmal tract of the fistula due to thrombosis; however, the flow in the native vessels was normal. Therefore, clopidogrel alone was continued to prevent potential thrombus extension proximally into the native coronary artery. After the procedure, her dyspnea disappeared, and she regained normal body weight after follow-up for 2 years.

## Discussion

Coronary artery fistulas are a rare disease accounting for 0.2–0.4% of congenital cardiac anomalies (6). CAFs arise most commonly from the RCA (55%), followed by the left coronary artery (35%), and rarely, 5% of fistulas are bilateral. Meanwhile, CAFs terminate mostly in the RV (45%), followed by the right atrium (25%), the pulmonary artery (15%), and less commonly the coronary sinus (7%) (3). An incomplete degeneration of sinusoidal connection between the lumens of primitive tubular heart in the early embryonic period may result in the formation of CAFs. The main hemodynamic drawback of abnormal shunts is coronary steal phenomenon and left-to-right shunt (6). Symptoms of CAFs depend on their size and severity of the shunt. Continuous heart murmur may be the only symptom in small shunts. However, large CAFs can result in heart failure, infective endocarditis, and myocardial ischemia (7).

The diagnosis of CAFs is confirmed by two-dimensional echocardiography and color Doppler sonography. Coronary artery dilatation is an important reminder of this disease as shown in our case (Figure 1A). Even though coronary angiography is essential to delineate the anatomy of CAFs, CTA can assist in non-invasive evaluation of CAFs before transcatheter intervention, especially in infants, to minimize the risk of catheterization. ECG and troponin tests are also helpful to detect myocardial ischemia.

The management of CAFs in children depends on the size and anatomy of the fistula and the presence of symptoms. According to American College of Cardiology/American Heart Association guidelines (8), treatment of small CAFs in asymptomatic patients is not suggested. Nonetheless, an intervention is recommended for large CAFs without symptoms and small to moderate-size fistulas with evidence of myocardial ischemia, arrhythmia, ventricular dysfunction, ventricular enlargement, or endarteritis (8). In our case, even though the Qp/Qs was small, this fistula is classified as large in size, since that fistulas, at any point larger than three times the expected proximate normal coronary artery diameter, or associated with similar ranges of dilation of the proximal associated coronary artery, are considered to be large size fistulas (9). In addition, coronary artery dilation and aneurysmal tract are often associated with stasis, which could lead to acute myocardial ischemia and aneurysmal rupture, respectively (10). After evaluating her clinical symptoms and fistula size and to prevent potential complications, we decided to perform percutaneous closure. As expected, her symptoms improved after the intervention.

In 1947, Biork et al. completed the first CAFs surgical closure (11). Since that procedure, surgical closure became the standard choice for closing CAFs. Even though the general outcome of surgical closure of CAFs is good, there is surgical mortality of 0–4% (12). CAF surgery is an invasive procedure that requires median sternotomy, and half of patients undergo cardiopulmonary bypass. Postoperative complications including bleeding, infections, myocardial infarctions, and arrhythmias were reported (11). The first CAF transcatheter closure was reported in 1983 by Reidy et al. Since then, transcatheter closure became an alternative option for closing a simple CAF, but surgery remains the only option for complex CAFs. The advantages of transcatheter closure of CAF over surgery are avoidance of cardiopulmonary bypass and median sternotomy, lower cost of the procedure, and shorter recovery time. The procedural complications of CAF transcatheter closure include device displacement, fistula dissection, myocardial infarction, and transient atrial arrhythmia (6). Recently, numerous reports of transcatheter closure have been described, with the successful closure rate of CAFs being around 75–87% (6). In general, the favorable anatomies for transcatheter closure of CAFs were single fistula, non-tortuous vessels, single narrow drainage site, and proximal origin of the fistula (13). Notwithstanding, in our case, the CAF drained into the RV in the distal part of the RCA, and the fistula was tortuous.

There are several important procedural aspects to be addressed in this case. First, the main challenge was to place the occluder with the antegrade approach from the RV *via* the femoral vein. In our patient, the retrograde approach of from the aorta to the RCA carried a high risk of complications such as myocardial ischemia, thrombosis, and aneurysmal rupture during occluder device delivery due to the tortuosity of the RCA and the distal drainage site. Thus, the antegrade method was chosen, and we established an arteriovenous wire loop from the tortuous RCA, through the aneurysmal fistula, to the RV. Then, a snare technique was used to catch the wire, and then the CAF was occluded. Second, procedures of occluding a CAF are more challenging in an infant because of the risk of vascular damage by the delivery sheath or the occluder device. In this context, we suggest that choosing a low-profile delivery system and a soft device has an important role to minimize potential complications. Various devices are used to close CAFs, such as coils, Amplatzer vascular plugs, ADO, and Rashkind double-umbrella devices (7, 14, 15). Among these devices, the ADO II has the following advantages in the context of CAFs with a narrow drainage site and an aneurysmal tract, especially in infants. The ADO II is made of a fabric-free fine nitinol wire resulting in its soft texture and can be delivered through a low-profile sheath. These features make it easier to pass through a narrow drainage site and safer to advance into the aneurysmal tract in the RV wall, with minimized risk of aneurysmal rupture or RV injury in an infant as shown in our case. Finally, the morphology of fistula in our patient was in a lageniform shape near the RV drainage site, which is also a favorable factor for using ADO II since it has two disks clamping both the RV wall and aneurysm, thus could have less chance of device dislodgement (11, 16, 17).

Anticoagulation after intervention was controversial. Low-dose aspirin (3–5 mg/kg/day) for at least 6 months was mentioned in most literature (18), and severe coronary artery dilatation (>10 mm) may warrant anticoagulation with warfarin (19). In our case, we prescribed a dual antiplatelet combination of aspirin and clopidogrel for 12 months followed by clopidogrel alone to prevent thrombosis in the dilated native RCA.

Long-term outcomes of transcatheter CAF closure from a limited case series demonstrate that this procedure is effective in most patients. However, complications can be found in certain patients at long-term follow-up, such as myocardial infarction and recanalization (20). Myocardial infarction can occur because of thrombus formation in the aneurysmal coronary artery or device thrombosis. In a case series, recanalization of a fistulous tract was found in 4 of 27 fistulas on repeat angiography at a median of 423 days after transcatheter closure (21). Thus, they recommended follow-up imaging study with coronary CT angiography or angiography for all patients who underwent successful CAF closure at 1–5 years to evaluate for recanalization.

## Conclusion

Transcatheter closure is an effective management for infants with large RCA to RV fistula with suitable anatomy. ADO II can be considered as the device of choice for infants with tortuous CAF.

## Data availability statement

The original contributions presented in the study are included in the article/supplementary material, further inquiries can be directed to the corresponding author.

## Author contributions

J-HH carried out the studies. Y-CL, Z-KD, and I-CC participated in collecting the data. S-HL drafted the manuscript. Y-HW helped to draft the manuscript. All authors contributed to the article and approved the submitted version.
